# Iron Reduces M1 Macrophage Polarization in RAW264.7 Macrophages Associated with Inhibition of STAT1

**DOI:** 10.1155/2017/8570818

**Published:** 2017-02-13

**Authors:** Zhen-Shun Gan, Qian-Qian Wang, Jia-Hui Li, Xu-Liang Wang, Yi-Zhen Wang, Hua-Hua Du

**Affiliations:** Key Laboratory of Animal Nutrition and Feed Science (Eastern of China), Ministry of Agriculture, College of Animal Science, Zhejiang University, Hangzhou 310058, China

## Abstract

Iron metabolism in inflammation has been mostly characterized in macrophages exposed to pathogens or inflammatory conditions. The aim of this study is to investigate the cross-regulatory interactions between M1 macrophage polarization and iron metabolism. Firstly, we characterized the transcription of genes related to iron homeostasis in M1 RAW264.7 macrophages stimulated by IFN-*γ*. The molecular signature of M1 macrophages showed high levels of iron storage (ferritin), a low level of iron export (ferroportin), and changes of iron regulators (hepcidin and transferrin receptors), which favour iron sequestration in the reticuloendothelial system and are benefit for inflammatory disorders. Then, we evaluated the effect of iron on M1 macrophage polarization. Iron significantly reduced mRNA levels of IL-6, IL-1*β*, TNF-*α*, and iNOS produced by IFN-*γ*-polarized M1 macrophages. Immunofluorescence analysis showed that iron also reduced iNOS production. However, iron did not compromise but enhanced the ability of M1-polarized macrophages to phagocytose FITC-dextran. Moreover, we demonstrated that STAT1 inhibition was required for reduction of iNOS and M1-related cytokines production by the present of iron. Together, these findings indicated that iron decreased polarization of M1 macrophages and inhibited the production of the proinflammatory cytokines. The results expanded our knowledge about the role of iron in macrophage polarization.

## 1. Introduction

Macrophages have long been considered to be important immune effector cells. Depending on the microenvironment, macrophages can acquire distinct morphological and functional properties. Different inflammatory stimuli can temporarily induce distinct subsets of macrophages with polarized inflammatory phenotypes. The Th1 cytokines such as IFN-*γ* stimulate the classic polarization and activation of macrophages into proinflammatory cells, which are often referred to as classically activated M1 macrophages [1]. M1 phenotype is characterized by high capacity to present antigen, high levels of inflammatory cytokines (TNF-*α*, IL-6) secretion and increased levels of NO production, enhanced capacity to kill intracellular pathogens and tumor cells, and promotion of polarized Th1 immune responses [2, 3]. Macrophages can also be alternatively activated by Th2 cytokines such as IL-4 and IL-13 and are characterized by minimal production of inflammatory molecules and wound healing and repair. These alternatively activated macrophages are referred to as M2 macrophages [4]. Generally, M1 macrophages are considered proinflammatory cells, whereas M2 macrophages are anti-inflammatory.

Macrophages also play a critical role in body iron homeostasis by recovering iron from old red blood cells and returning it to the circulation. They are prodigious phagocytic cells that clear approximately 2 × 10^11^ erythrocytes each day, which equate almost 3 kg of iron and hemoglobin per year that is “recycled” for the host to reuse [5, 6]. Iron is an essential trace element for multicellular organisms and nearly all microorganisms, in which it functions as a catalytic component of enzymes that mediate many redox reactions that are crucial for energy production and intermediary metabolism [7]. Iron retention in the reticuloendothelial system is the main response of body iron homeostasis to inflammation and is regarded as a host's attempt to withhold iron from the invading pathogens [8]. Increased iron retention within inflammatory macrophages is due to increased iron uptake and decreased iron export and is favoured by the induction of the iron storage protein ferritin (Ft) [9, 10].

So far, cytokines, which drive macrophage polarization, have been reported to ultimately control iron handling [11, 12]. However, there is limited information about the effect of iron on the polarization and function of M1 macrophages. In the present study, we characterized the changes in iron trafficking in M1 macrophages and investigated the effects of iron on M1 polarization of RAW264.7 macrophage stimulated by interferon-gamma (IFN-*γ*). We found that iron dramatically inhibited the transcription of proinflammatory cytokine and the production of enzyme inducible nitric oxide synthase (iNOS) in M1 macrophages. We further demonstrated that iron decreased M1 macrophage polarization involves inhibition of signal transducer and activator of transcription 1 (STAT1) pathway.

## 2. Materials and Methods

### 2.1. Reagents

Ferric ammonium citrate (FAC) was purchased from Sigma (USA). Recombinant murine interferon-gamma (IFN-*γ*) was purchased from PeproTech (USA).

### 2.2. Cell Culture and Stimulation

The RAW264.7 macrophage cell line was generously offered by professor WeifenLi's Laboratory (College of Animal Science, Zhejiang University, Hangzhou, China). RAW264.7 cells were cultured in DMEM with 10% FBS (Gibco, USA), 100 U/mL penicillin, and 100 *μ*g/mL streptomycin at 37°C in 5% CO_2_ in humidified incubator. In a 6-well bottom plate, 1 × 10^6^ cells per well were seeded and incubated at 37°C for 12 h. The RAW264.7 cells were stimulated with FAC (0~400 *μ*g/mL) for 24 h and the cytotoxicity assays were performed to ensure the appropriate FAC concentration. The RAW264.7 macrophage cells were treated with 25 *μ*g/mL FAC or IFN-*γ* (20 ng/mL) with or without FAC for 24 h, which were used for gene expression, western blot analysis, and immunofluorescence or phagocytosis test.

### 2.3. Cytotoxicity Assay

Cell proliferation assay was evaluated by a Cell Counting Kit-8 (CCK-8) Kit (Dojindo, Japan). Briefly, monolayers of RAW264.7 cells in 96-well microplate were cultured in DMEM supplemented with 10% FBS and incubated with FAC (3.125~400 *μ*g/mL) for 24 h. The medium was replaced with fresh DMEM containing CCK-8. CCK-8, being nonradioactive, allows sensitive colorimetric assays for the determination of the number of viable cells in cell proliferation and cytotoxicity assays. After 2 h of incubation, the optical density was measured at OD_450_. Lactate dehydrogenase (LDH) release from damaged cells was determined 48 h after treatment with FAC (3.125~400 *μ*g/mL). LDH activity in the culture supernatant was measured using a Cytotoxicity LDH Assay Kit-WST (Dojindo, Japan).

### 2.4. Total RNA Isolation and Real-Time PCR

Total RNA isolated from RAW264.7 cells was reverse transcribed using MMLV Reverse Transcriptase (Thermo Fisher Scientific, USA). Real-time PCR was performed using FastStrat Universal SYBR Green Master (ROX) (Roche, USA) and the ABI 7500 real-time PCR system (Applied Biosystems). The following primers were used: Hamp2 forward 5′-ATCCCAATGCAGAAGAGAAGG-3′ and reverse 5′-CAGATACCACAGGAGGGTTTG-3′; FPN forward 5′-GGGTGGATAAGAATGCCAGACTT-3′ and reverse 5′-GTCAGGAGCTCATTCTTGTGTAGGA-3′; FtH forward 5′-TGGAACTGCACAAACTGGCTACT-3′ and reverse 5′-ATGGATTTCACCTGTTCACTCAGATAA-3′; FtL forward 5′-CGTGGATCTGTGTCTTGCTTCA-3′ and reverse 5′-GCGAAGAGACGGTGCAGACT-3′; IRP1 forward 5′-ACTTTGAAAGCTGCCTTGG-3′ and reverse 5′-CTCCACTTCCAGGAGACAGG-3′; IRP2 forward 5′-TGAAGAAACGGACCTGCTCT-3′ and reverse 5′-GCTCACATCCAACCACCTCT-3′; IL-6 forward 5′-CTCCGACTTGTGAAGTGGTATAG-3′ and reverse 5′-CCACCTCAATGGACAGAATATCA-3′; IL-1*β* forward 5′-AGTTGACGGACCCCAAAAG-3′ and reverse 5′-TTTGAAGCTGGATGCTCTCAT-3′; TNF-*α* forward 5′-GCTCTTCTGTCTACTGAACTTCGG-3′ and reverse 5′-ATGATCTGAGTGTGAGGGTCTGG-3′; iNOS forward 5′-CAGCTGGGCTGTACAAACCTT-3′ and reverse 5′-CATTGGAAGTGAAGCGTTTCG-3′; *β*-actin forward 5′-CCACCATGTACCCAGGCATT-3′ and reserse 5′-AGGGTGTAAAACGCAGCTCA-3′. Fold changes were calculated after normalizing the change in expression of the gene of interest to the housekeeping gene *β*-actin using the threshold cycle values.

### 2.5. Western Blot Analysis

Total cell protein was prepared using a Whole Protein Extraction Kit (KeyGEN, China). Protein concentrations were determined using a BCA Assay Kit (KeyGEN, China). Equal amounts of proteins from each sample were subjected to SDS-PAGE followed by transfer of proteins to polyvinylidene difluoride (PVDF) membranes. Membranes were blocked in 5% skimmed milk and incubated with a primary antibody overnight at 4°C. After washing with TBST, membranes were incubated with secondary antibody linked to HRP. The blots were then developed with an ECL detection system (Santa Cruz, USA).

### 2.6. Immunofluorescence Analysis

iNOS protein expression levels of the RAW264.7 cells were evaluated by confocal immunofluorescence microscopy. Briefly, the RAW264.7 cells were incubated with a rabbit monoclonal anti-iNOS antibodies (Abcam, USA) for overnight at 4°C and then with goat anti-rabbit IgG/Cy3 secondary antibodies for 1 h. After washing with PBS, the cells were incubated in a medium containing 40 mg/ml DAPI for 5 min and examined with a laser-scanning microscope (ZEISS, Germany).

### 2.7. Phagocytosis Assay

To analyze the phagocytic activity of macrophages, The RAW264.7 macrophage cells were polarized by IFN-*γ* (20 ng/mL) in the presence of 25 *μ*g/mL FAC for 24 h and then incubated with fluorescein isothiocyanate- (FITC-) dextran (1 mg/mL) at 37°C for 1 h. After incubation, the cells were washed twice with PBS and the percentage of intracellular FITC-dextran was determined by Fluorescence Activating Cell Sorter (FACS).

### 2.8. Data Analysis

The data were expressed as the mean ± SD of three independent experiments. Statistical analyses were performed using two-tailed Student's *t*-test. Values of *p* < 0.05 were considered significant.

## 3. Results

### 3.1. Differentially Expressed Genes of Iron Metabolism in M1 Macrophages

**Figure 1:**
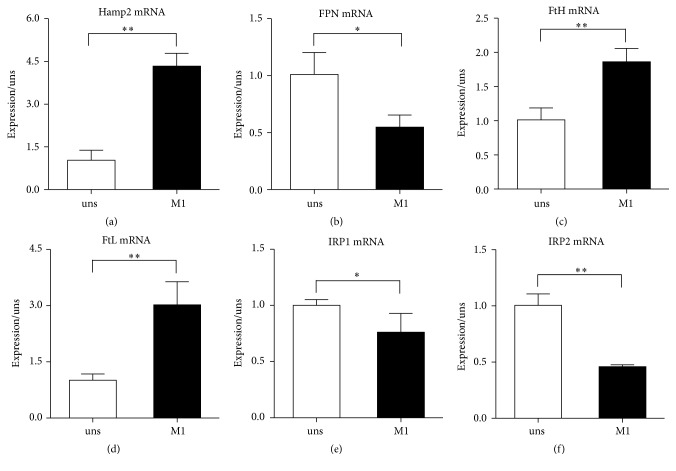
Differential expression of iron metabolism-related genes in M1 macrophages. (a) Hamp2, (b) FPN, (c) FtH, (d) FtL, (e) IRP1, and (f) IRP2 mRNA levels in 24 h cells after polarization. The expression was normalized to *β*-actin and then expressed in relation to unstimulated macrophages, arbitrarily defined as 1. Data are mean ± SD for three independent experiments. (uns: unstimulated macrophages; ^*∗*^*p* < 0.05, ^*∗∗*^*p* < 0.01).

We relied on established protocols to polarize RAW264.7 macrophages into M1 cells by exposure to IFN-*γ* [13]. Polarization of macrophages skews the expression profile of genes involved in iron metabolism. In comparison with unstimulated macrophages, the transcript levels of hepcidin (Hamp2), which is the master regulator of iron homeostasis, were increased in M1 cells (Figure 1(a)). Accordingly, the transcripts of ferroportin (FPN), which is the main and possibly exclusive iron exporter and is functionally involved in modulating iron release, were decreased in M1 cells (Figure 1(b)). The mRNA levels of ferritin heavy chain (FtH) and ferritin light chain (FtL), which are associated with iron storage, were increased in IFN-*γ* stimulated macrophages (Figures 1(c)-1(d)). We also analyzed iron regulatory proteins (IRP1 and IRP2), which are proteins of iron metabolism important in intracellular iron homeostasis and known to be primarily regulated at the posttranscriptional level [14]. Lower mRNA expressions of IRP1 and IRP2 were detected in cells exposed to IFN-*γ* (Figures 1(e)-1(f)).

### 3.2. Determination of the Noncytotoxic Dose of Iron in Macrophages

**Figure 2:**
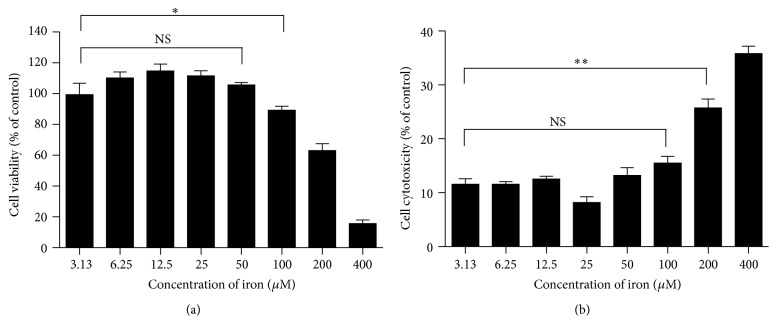
Determination of the noncytotoxic dose of iron. (a) RAW264.7 macrophage cells were incubated with FAC at range from 0 to 400 *μ*g/mL for 24 h. Cell viability was determined by CCK-8 method. The results are expressed as the percentage of viable cells and represent mean ± SD of four samples. (b) Cell death was confirmed by measuring the release of the cytosolic marker LDH. LDH activity in the supernatant was measured as described in methods. ^*∗*^*p* < 0.05 and ^*∗∗*^*p* < 0.01 (*t*-test).

We evaluated the cytotoxicity of iron (FAC) ranging from 3.13 to 400 *μ*g/mL on RAW264.7 cells and found that the optimal viability was 25 *μ*g/mL, showing 100% survival (Figure 2(a)). Moreover, we confirmed cell damage by measuring the release of the cytosolic marker lactate dehydrogenase (LDH). Treatment with less than 100 *μ*g/mL FAC for 48 h showed no significant difference of LDH release when compared with the control group (Figure 2(b)). Therefore, 25 *μ*g/mL FAC was used for the next experiments.

### 3.3. Iron Reduced the Transcription of Proinflammatory Mediators by M1-Polarized Macrophage

**Figure 3:**
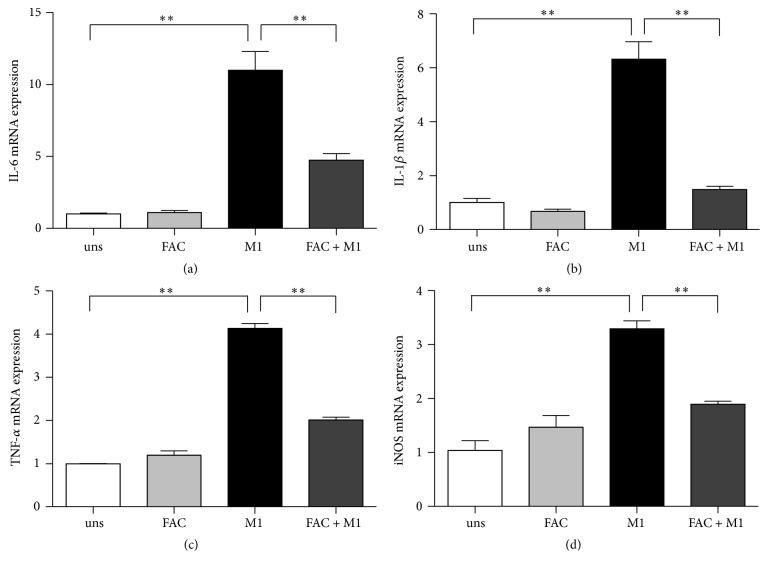
Effects of iron on mRNA expression in M1 macrophage. (a) IL-6, (b) IL-1*β*, (c) TNF-*α*, and (d) iNOS mRNA expression were assessed by real-time PCR. Gene expression is represented as fold-change compared to unstimulated macrophages. Data are mean ± SD for three independent experiments. (uns: unstimulated macrophages; ^*∗∗*^*p* < 0.01).

As transcriptions have been shown to be a major mechanism in monocytic cells for LPS or IFN-*γ* stimulation, we used qRT-PCR to determine if iron was affecting the transcription of cytokines. Proinflammatory M1 RAW264.7 macrophages increased high mRNA levels of IL-6, IL-1*β*, TNF-*α*, and iNOS in response to the stimulation of IFN-*γ*, whereas unstimulated macrophages produced significantly lower levels (Figure 3). The addition of 25 *μ*g/ml FAC during the polarization of M1 macrophages by IFN-*γ* resulted in a severe blockage of the transcription of cytokines production (Figure 3). IL-1*β* mRNA was significantly inhibited by 77% (Figure 3(b)). FAC reduced IL-6 mRNA by 57% (Figure 3(a)) and TNF-*α* mRNA by 51% (Figure 3(c)). It had a much lesser effect on iNOS production, although it still significantly reduced the transcription by 43% (Figure 3(d)).

### 3.4. Iron Inhibited iNOS Production in M1 Macrophages

**Figure 4:**
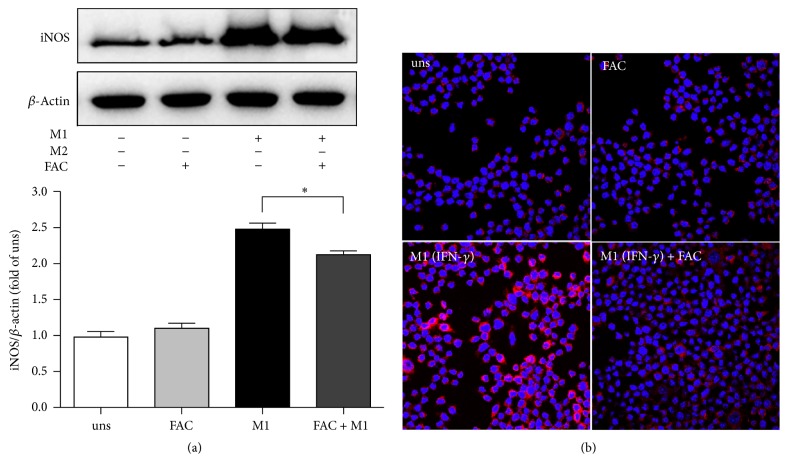
Effects of iron on iNOS expression in M1 macrophage. RAW264.7 macrophage cells were incubated with the stimulus, either FAC, IFN-*γ*, or no stimulus for 24 h. Cells were harvested and analyzed for iNOS (a) by western blot or fixed and stained with antibodies for iNOS (b). Protein expression is represented as fold-change compared to unstimulated macrophages. In each sample group, cell was stained with the red fluorescent-labeled antibody for the targeted protein and the blue fluorescent-labeled DAPI for nucleus. Data are representative images of three (a) or two (b) independent experiments. (uns: unstimulated macrophages; ^*∗*^*p* < 0.05).

M1 macrophages produce inducible nitric oxide synthase (iNOS) that enables the cell to kill intracellular pathogens through the production of NO. We further investigated whether iron regulates iNOS production. In line with the results of transcription (Figure 3(d)), the M1-polarized macrophages produced higher iNOS in response to IFN-*γ* compared with unstimulated cells. However, the presence of 25 *μ*g/ml FAC during the polarization resulted in a blockage of iNOS production with 10% inhibition (Figure 4(a)). To confirm the outcomes at a protein level and to determine if the changes in protein expression have occurred uniformly across the entire macrophage population, cells were stimulated by IFN-*γ* in the presence of 25 *μ*g/ml FAC and examined by immunofluorescence microscopy with antibody specific for iNOS. Consistent with mRNA expression outcomes (Figure 3(d)), cells stimulated with IFN-*γ* expressed higher level of iNOS (Figure 4(b), bottom left side, red). The presence of FAC significantly blocked the production of iNOS (Figure 4(b), bottom right side, red). Of note, staining levels were uniform in most cells within the same condition, indicating that the mRNA or protein expression patterns result from changes across the entire populations.

### 3.5. Iron Inhibited STAT1 Pathway in M1 Macrophages

**Figure 5:**
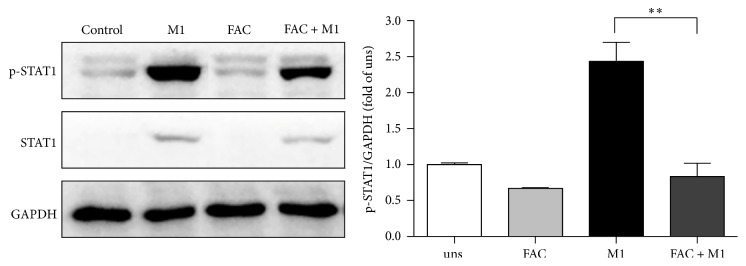
Effect of iron on STAT1 activation in M1 macrophages. RAW264.7 macrophage cells were incubated with the stimulus, either FAC, IFN-*γ*, or no stimulus for 24 h. Cell lysates were prepared, and phosphorylation of STAT1 (p-STAT1) was analyzed by western blot. One representative blot and the densitometric quantification are shown. Data are mean ± SD for three independent experiments. (uns: unstimulated macrophages; ^*∗∗*^*p* < 0.01).

Macrophage polarization is a complex process including stimuli recognition and activation of the transcription factors [15]. Recent studies have shown that STAT1 signaling pathways are involved in M1 macrophage polarization [16]. To investigate whether iron affects these cascades, we performed western blot to examine the phosphorylation of STAT1. M1-polarized macrophages increased the phosphorylation forms of STAT1 in response to IFN-*γ* compared with unstimulated cells. However, the presence of 25 *μ*g/ml FAC during the polarization resulted in a blockage the phosphorylation of STAT1 with 66% inhibition (Figure 5), indicating that FAC decreased M1 macrophage polarization was dependent on STAT1 signaling.

### 3.6. Iron Enhanced Phagocytosis Capacity of M1 Phagocytosis

**Figure 6:**
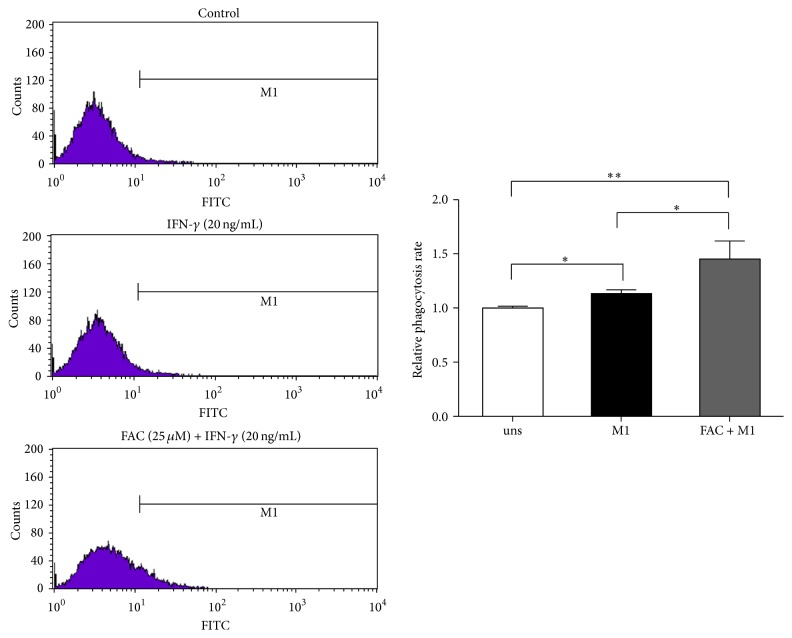
Effect of iron on M1 macrophages phagocytosis activity. RAW264.7 macrophages were polarized to an M1 phenotype in the absence (uns) or presence of 25 *μ*g/mL of FAC, washed, and incubated with FITC-dextran at 37°C for 1 h; the intracellular FITC-dextran was measured by FACS. Data are mean ± SD for three independent experiments. (uns: unstimulated macrophages; ^*∗*^*p* < 0.05, ^*∗∗*^*p* < 0.01).

Phagocytosis plays a crucial role in macrophage-mediated host defense, which leads to internalization and distraction of pathogens. To determine if iron affected the phagocytosis of M1-polarized macrophages, we examined the internalization of FITC-labeled dextran by FACS. FAC-treated M1 macrophages showed markedly increased uptake of FITC-dextran (Figure 6). Therefore, iron inhibits the production of the proinflammatory cytokines in M1 macrophages without affecting their phagocytosis functions.

## 4. Discussion

Macrophages are important for immune responses and are widely distributed in peripheral tissues where they play an indispensable role in the defense against pathogens. This is at least partially achieved through the control of intracellular iron availability, which limits pathogen growth [17]. As an essential trace element for microbes proliferation and pathogenicity, iron affects cell-mediated immune function and thus host response toward pathogens. On the one hand, the polarization of macrophages can have important effects on iron metabolism, but on the other hand iron can influence directly macrophage polarization [18]. The aim of this study is to investigate the cross-regulatory interactions between M1 macrophage polarization and iron metabolism.

Cellular iron homeostasis in macrophages is regulated at multiple steps and by numerous genes [19]. Macrophages can acquire iron via the divalent metal transporter 1 and phagocytosis of senescent erythrocytes with subsequent recycling of iron. The diversion of cellular iron is then orchestrated by the IRP/IRE interaction resulting in reutilization, iron storage within ferritin, or iron export. FPN is only one well-characterized pathway for iron export from cells [20]. Hepcidin, induced by iron and cytokines and master regulator of body iron homeostasis, exerts its regulatory effects via binding to its receptor FPN [21]. The transcriptional and posttranscriptional control of many of the genes are responsible for these functions [13], so we detected the mRNA levels of iron related genes to characterize the changes of iron metabolism in M1 macrophages. In this study, M1 macrophages derived by recombinant IFN-*γ* alone express high mRNA levels of hepcidin (Hamp2), FtH, and FtL and low levels of FPN, IRP1, and IRP2. M1 macrophages, which directly deal with microbes at sites of infection, upregulate hepcidin and downregulate FPN, thus limiting release of iron which could favour invading pathogens. Meanwhile, by upregulating FtH and FtL expression and limiting IRP1 and IRP2, M1 cells possibly protect themselves against oxidative damage and further limit the availability of the molecule to internalized microbes. These observations are consistent with experiments on human peripheral blood monocytes and mice macrophages [11, 12].

It is well known that M1 macrophages have enhanced microbicidal capacity, secrete high levels of proinflammatory cytokines, and produce great amount of oxygen and nitrogen radicals to increase their killing activity [22]. In this study, we found that iron dramatically inhibited the transcriptions of proinflammatory cytokine IL-6, IL-1*β*, and TNF-*α* in IFN-*γ*-stimulated M1 macrophages. Proinflammatory cytokines, as markers of activated polarized M1 macrophages, are regarded as the effector molecules to mediate resistance against pathogens [16]. Prevention of the transcription of these proinflammatory cytokines indicated that a major effect of iron is to prevent IFN-*γ*-induced activation of these genes. Meanwhile, M1 macrophages produce high levels of iNOS, which is a major component of the antimicrobial effector machinery, and the formation of NO has been shown to mediate protection from infection [23]. We observed that iron also had a mild suppressive effect on iNOS production, which led to a decreased production of NO. Iron might inhibit the expression of iNOS by a transcriptional mechanism involving the deactivation of the transcription factor nuclear factor (NF)-IL-6 [24]. However, the potent inhibitory effect of iron was restricted to specific functions of classically activated macrophages, namely, proinflammatory cytokines and iNOS production, as it did not affect the phagocytosis capacity of M1 macrophages.

STAT1 is an important signaling molecule that plays a major role in mediating proinflammatory responses following ligation of the Th1-type cytokine IFN-*γ*, which is important for modulating protective immune responses to multiple pathogens [25]. Here, we showed that iron also decreased the phosphorylation of STAT1 in IFN-*γ*-induced macrophages, which suggested that STAT1 inhibition might be required for reduction of iNOS and M1-related cytokines production. This result was in line with previous report that STAT1 signaling in macrophages during* C. neoformans* infection is critical for the induction of M1 macrophage activation and the production of NO [26].

Iron exerts multiple effects on immune effector functions. This is on the one hand based on the role of iron for the differentiation and proliferation of immune cells, including antigen presenting cells and lymphocytes [27]. Moreover, iron affects antimicrobial immune function of macrophages via inhibition of IFN-*γ* inducible effector pathways [28, 29]. The relevance of these observations was substantiated by experiments, which demonstrated that macrophages loaded with iron lose their ability to kill intracellular pathogens such as* Salmonella*,* Mycobacteria*,* Chlamydia*, or* Legionella *by IFN-*γ*-mediated pathways [17, 30, 31]. To the best of our knowledge, there is not previous report on the effect of iron addition on the macrophage polarization. Our data provide evidence that iron inhibits the polarization of M1 macrophages stimulated by IFN-*γ* and then impairs the proinflammatory responses of macrophages.

Obviously, the retention of iron in the M1 macrophages reduces circulating iron levels and thus the availability of this essential nutrient for extracellular microbes. This iron withholding strategy appears to be of benefit to combat infections with circulating pathogens. Thus, iron supplementation during infections is inadvisable and even hazardous. Clinical trials demonstrated that iron supplementation resulted in higher incidence of or higher mortality from infections such as malaria, diarrhea, or bacterial meningitis [32, 33]. The pathways underlying these devastating outcomes remain elusive. However, they may be linked to iron mediated modulation of antimicrobial immune defense of macrophages or traced back to increased availability of the metal for pathogens.

In conclusion, based on discovering iron sequestration in M1 macrophages, we have demonstrated that iron suppressed IFN-*γ* induced M1 polarization of RAW264.7 macrophages with decreased proinflammatory responses and iNOS production, while preserving their phagocytosis activity. It suggested that iron loading during M1 polarization would impair the antimicrobial immune defense of macrophages. Thus, a certain balance of iron, not too less and not too much, is needed to strengthen immune response to successfully combat infections.
